# Supplementary material for: Predictive model identifies strategies to enhance TSP1-mediated apoptosis signaling
Source: Cell Commun Signal. 2017 Dec 19;15:53. doi: 10.1186/s12964-017-0207-9 (PMC5735807; doi:10.1186/s12964-017-0207-9)
Supplement: Supplementary file 2 — Ordinary differential equations and initial conditions (PDF 47 kb) [file 12964_2017_207_MOESM2_ESM.pdf]

```
%% Initial Conditions
TSP1 = y(1);
CD36 = y(2);
TSP1_CD36 = y(3);
p59 = y(4);
TSP1_CD36_p59 = y(5);

pro8 = y(6);
casp8 = y(7);
pro3 = y(8);
casp3 = y(9);
p38cyt = y(10);

pp38cyt = y(11);
Ptase_cyt = y(12);
pp38cyt_Ptase = y(13);
pp59_Ptase = y(14);
XIAP = y(15);

PARP = y(16);
casp3_PARP = y(17);
cPARP = y(18);
casp3_u = y(19);
FasL_cyt = y(20);

FasLm_cyt = y(21);
Fas = y(22);
casp3_MEKK1 = y(23);
cMEKK1 = y(24);
MEKK1 = y(25);

cMEKK1_p38cyt = y(26);
FL = y(27);
DISC = y(28);
FasL = y(29);
DISC_pro8 = y(30);

DISC_FL = y(31);
FS = y(32);
DISC_FS = y(33);
p43p41 = y(34);
p43_FLIP = y(35);

DISC_pro8_FS = y(36);
DISC_FL_FL = y(37);
DISC_FL_FS = y(38);
DISC_FS_FS = y(39);
IKK = y(40);

p43_FLIP_IKK_a = y(41);
NFkB_IkB = y(42);
NFkB_IkB_p = y(43);
NFkB_cyt = y(44);
TSP1_CD36_pp59 = y(45);

pp38nuc = y(46);
p38nuc = y(47);
Ptase_nuc = y(48);
pp38nuc_Ptase = y(49);
FasLm_nuc = y(50);

NFkB_nuc = y(51);
pp38_NFkB = y(52);
```

```
NFkB_p = y(53);

%% Parameters
Kon_dephos = params(1,1);
Kdephos = params(2,1);
Ki_casp3_XIAP = params(3,1);
Ktrsp_cyt_nuc = params(4,1);
Kinter = params(5,1);

Vmax_FasLm = params(6,1);
Km_FasLm = params(7,1);
Kexp_FasLm = params(8,1);
Ksyn_FasL = params(9,1);
Kexp_FasL = params(10,1);

Kon_Fas_FasL = params(11,1);
Kon_DISC_pro8 = params(12,1);
Ksyn_all = params(13,1);
Kdeg = params(14,1);
Kon_casp3_MEKK1 = params(15,1);


Kp_p38 = params(16,1);
Kc_MEKK1 = params(17,1);
Kon_cMEKK1_p38 = params(18,1);
FasLsyn_factor = params(19,1);
Kon_DISC_FL = params(20,1);

Kon_DISC_FS = params(21,1);
Kp43p41 = params(22,1);
Kp43FLIP = params(23,1);
Kon_DISC2_FS = params(24,1);
K_casp8 = params(25,1);

Kc_casp3 = params(26,1);
Kr_casp8 = params(27,1);
Ka_IKK = params(28,1);
Kp_NFkBIkB = params(29,1);
Ka_NFkB = params(30,1);

Kdeg_IKKa = params(31,1);
Kdeg_NFkBcyt = params(32,1);
Kdeg_casp8 = params(33,1);
Kdeg_casp3 = params(34,1);
Kon_pp38_NFkB = params(35,1);

Kdeg_pp38cyt = params(36,1);
Kon_TSP1_CD36 = params(37,1);
Kon_TSP1_CD36_p59 = params(38,1);
Kp_p59 = params(39,1);
Kp_NFkB = params(40,1);

Kon_casp3_PARP = params(41,1);
Kc_PARP = params(42,1);
Ksyn_Ptase = params(43,1);
Kdeg_Ptase = params(44,1);
Kc_casp8 = params(45,1);

Kinser = params(46,1);
koff= params(47,1);
Koff_dephos = params(48,1);
Koff_casp3_MEKK1 = params(49,1);
Koff_cMEKK1_p38 = params(50,1);
```

```
Koff_pp38_NFkB = params(51,1);
Koff_TSP1_CD36 = params(52,1);
Koff_TSP1_CD36_p59 = params(53,1);
Koff_casp3_PARP = params(54,1);

cytosol = 0.824;
nucleus = 0.118;
mc = 0.058;

%% Fluxes
RF1 = Kon_Fas_FasL*FasL*Fas-koff*DISC;
RF2 = Kon_DISC_pro8*DISC*pro8-koff*DISC_pro8;
RF3 = Kp43p41*DISC_pro8*pro8;
RF4 = Kon_DISC_FS*FS*DISC-koff*DISC_FS;
RF5 = Kdeg_casp8*casp8;

RF6 = Kdeg_casp3*casp3;
RF7 = Kon_DISC_FL*DISC*FL-koff*DISC_FL;
RF8 = Kp43FLIP*DISC_pro8*FL;
RF9 = Kon_DISC2_FS*DISC_pro8*FS-koff*DISC_pro8_FS;
RF10 = Kp43p41*DISC_FL*pro8;

RF11 = Kp43FLIP*DISC_FL*FL-koff*DISC_FL_FL;
RF12 = Kon_DISC2_FS*DISC_FL*FS-koff*DISC_FL_FS;
RF13 = Kp43p41*DISC_FS*pro8-koff*DISC_pro8_FS;
RF14 = Kp43FLIP*DISC_FS*FL-koff*DISC_FL_FS;
RF15 = Kon_DISC2_FS*DISC_FS*FS-koff*DISC_FS_FS;

RF16 = K_casp8*p43p41*p43p41;
RF17 = Kc_casp3*casp8*pro3;
RF18 = Kr_casp8*casp3*pro8;
RF19 = Ka_IKK*p43_FLIP*IKK;
RF20 = Kp_NFkBIkB*NFkB_IkB*p43_FLIP_IKK_a;

RF21 = Ka_NFkB*NFkB_IkB_p;
RF22 = Kdeg_IKKa*p43_FLIP_IKK_a;
RF23 = Kdeg_NFkBcyt*NFkB_cyt;
RF24 = Kon_TSP1_CD36*TSP1*CD36 - Koff_TSP1_CD36*TSP1_CD36;
RF25 = Kon_TSP1_CD36_p59*TSP1_CD36*p59 - Koff_TSP1_CD36_p59*TSP1_CD36_p59;

RF26 = Kp_p59*TSP1_CD36_p59;
RF27 = Kc_casp8*TSP1_CD36_pp59*pro8;
RF28 = K_casp8*p43p41*p43p41;
RF29 = Kc_casp3*casp8*pro3;
RF30 = Kon_casp3_MEKK1*casp3*MEKK1 - Koff_casp3_MEKK1*casp3_MEKK1;

RF31 = Kc_MEKK1*casp3_MEKK1;
RF32 = Kon_cMEKK1_p38*cMEKK1*p38cyt - Koff_cMEKK1_p38*cMEKK1_p38cyt;
RF33 = Kp_p38*cMEKK1_p38cyt;
RF34 = Ktrsp_cyt_nuc*p38cyt;
RF35 = Ktrsp_cyt_nuc*pp38cyt;

RF36 = Ktrsp_cyt_nuc*pp38cyt*cytosol/nucleus;
RF37 = Ktrsp_cyt_nuc*p38cyt*cytosol/nucleus;
RF38 = Kon_pp38_NFkB*pp38nuc*NFkB_nuc - Koff_pp38_NFkB*pp38_NFkB;
RF39 = Kp_NFkB*pp38_NFkB;
RF40 = Vmax_FasLm*NFkB_p/(Km_FasLm+NFkB_p);


RF41 = Kexp_FasLm*FasLm_nuc;
RF42 = Kexp_FasLm*FasLm_nuc*nucleus/cytosol;
RF43 = Ksyn_FasL*FasLm_cyt;
```

RF44 = Kexp_FasL*FasL_cyt;
RF45 = Kon_casp3_PARP*casp3*PARP - Koff_casp3_PARP*casp3_PARP;

RF46 = Ki_casp3_XIAP*casp3*XIAP;
RF47 = Kc_PARP*casp3_PARP;
RF48 = Kon_dephos*TSP1_CD36_pp59*Ptase_cyt - Koff_dephos*[pp59_Ptase];
RF49 = Kon_dephos*pp38cyt*Ptase_cyt - Koff_dephos*[pp38cyt_Ptase];
RF50 = Kon_dephos*pp38nuc*Ptase_nuc- Koff_dephos*[pp38nuc_Ptase];

RF51 = FasLsyn_factor*DISC + Ksyn_all;
RF52 = FasLsyn_factor*DISC + Ksyn_all;
RF53 = Ktrsp_cyt_nuc*NFkB_cyt;
RF54 = Kdephos*pp59_Ptase;
RF55 = Kdephos*pp38cyt_Ptase;

RF56 = Kdephos*pp38nuc_Ptase;
RF57 = Ksyn_all;
RF58 = Ksyn_all;
RF59 = Ksyn_all;
RF60 = Ksyn_all;

RF61 = Ksyn_all;
RF62 = Ksyn_all;
RF63 = Ksyn_all;
RF64 = Ksyn_all;
RF65 = Ksyn_all;

RF66 = Ksyn_all;
RF67 = Ksyn_all;
RF68 = Ksyn_Ptase;
RF69 = Ksyn_Ptase;
RF70 = Kdeg_Ptase*Ptase_nuc;

RF71 = Kdeg_Ptase*Ptase_cyt;
RF72 = Kdeg*CD36;
RF73 = Kdeg*TSP1_CD36;
RF74 = Kdeg*TSP1_CD36_p59;
RF75 = Kdeg*TSP1_CD36_pp59;

RF76 = Kdeg*p43p41;
RF77 = Kdeg*casp3_MEKK1;
RF78 = Kdeg*cMEKK1;
RF79 = Kdeg*casp3_PARP;
RF80 = Kdeg*PARP;

RF81 = Kdeg*XIAP;
RF82 = Kdeg*casp3_u;
RF83 = Kdeg*p38cyt;
RF84 = Kdeg*cMEKK1_p38cyt;
RF85 = Kdeg_pp38cyt*pp38cyt;

RF86 = Kdeg*pp38nuc;
RF87 = Kdeg*p38nuc;
RF88 = Kdeg*Fas;
RF89 = Kdeg*DISC;
RF90 = Kdeg*DISC_FS;

RF91 = Kdeg*DISC_FL;
RF92 = Kdeg*DISC_pro8;
RF93 = Kdeg*p43_FLIP;
RF94 = Kdeg*DISC_FL_FL;
RF95 = Kdeg*DISC_FL_FS;

```
RF96 = Kdeg*DISC_FS_FS;
RF97 = Kdeg*IKK;
RF98 = Kdeg*NFkB_IkB;
RF99 = Kdeg*NFkB_IkB_p;
RF100 = Kdeg*pp59_Ptase;

RF101 = Kdeg*pp38cyt_Ptase;
RF102 = Kdeg*pp38nuc_Ptase;
RF103 = Kdeg*NFkB_nuc;
RF104 = Kdeg*pp38_NFkB;
RF105 = Kdeg*NFkB_p;

RF106 = Ktrsp_cyt_nuc*NFkB_cyt*cytosol/nucleus;
RF107 = Kdeg*DISC_pro8_FS;
RF108 = Kinter*CD36;
RF109 = Kinter*TSP1_CD36;
RF110 = Kinter*TSP1_CD36_p59;

RF111 = Kinter*TSP1_CD36_pp59;
RF112 = Kinser;
RF113 = Kinter*Fas;
RF114 = Kinter*DISC;
RF115 =Kinter*DISC_FL;

RF116 =Kinter*DISC_FS;
RF117 =Kinter*DISC_pro8;
RF118 =Kinter*DISC_FL_FL;
RF119 =Kinter*DISC_FL_FS;
RF120 =Kinter*DISC_FS_FS;

RF121 =Kinter*DISC_pro8_FS;
RF122 = Kdeg*p59;
RF123 = Kdeg*pro8;
RF124= Kdeg*pro3;
RF125 = Kdeg*FasL_cyt;

RF126 = Kdeg*MEKK1;
RF127 = Kdeg*FL;
RF128 = Kdeg*FS;

%% ODEs

dydt(1,1) = -RF24;%TSP1
dydt(2,1) = -RF24 + RF57 - RF72-RF108 + RF112*0.0332;%CD36
dydt(3,1) = RF24 - RF25 - RF73-RF109;%TSP1_CD36
dydt(4,1) = -RF25 + RF58 - RF122;%p59
dydt(5,1) = RF25 - RF26 + RF54 - RF74-RF110;%TSP1_CD36_p59

dydt(6,1) = -RF2 - RF3 - RF10 - RF13 - RF18 - RF27 + RF52 - RF123;%pro8
dydt(7,1) = -RF5 + RF16 + RF27 + RF28;%casp8
dydt(8,1) = -RF17 - RF29 + RF51 - RF124;%pro3
dydt(9,1) = -RF6 + RF17 + RF29 - RF30 + RF31 - RF45 - RF46 + RF47;%casp3
dydt(10,1) = -RF32 - RF34 + RF55 + RF59 - RF83;%p38cyt

dydt(11,1) = RF33 - RF35 - RF49 - RF85;%;%pp38cyt
dydt(12,1) = -RF48 - RF49 + RF54 + RF55 + RF68 - RF71;%Ptase_cyt
dydt(13,1) = RF49 - RF55 - RF101;%pp38cyt_Ptase
dydt(14,1) = RF48 - RF54 - RF100;%pp59_Ptase
dydt(15,1) = -RF46 + RF60 - RF81;%XIAP

dydt(16,1) = -RF45 + RF61 - RF80;%PARP
dydt(17,1) = RF45 - RF47 - RF79;%casp3_PARP
dydt(18,1) = RF47-Ksyn_all*cPARP;%cPARP
```

```
dydt(19,1) = RF46 - RF82;%;%casp3_u
dydt(20,1) = RF43 - RF44 - RF125;%FasL_cyt

dydt(21,1) = RF42 - RF43;%FasLm_cyt
dydt(22,1) = -RF1 + RF62 - RF88+RF112*0.0149-RF113;%Fas
dydt(23,1) = RF30 - RF31 - RF77;%casp3_MEKK1
dydt(24,1) = RF31 - RF32 + RF33- RF78;%cMEKK1
dydt(25,1) = - RF30 + RF63 - RF126;%MEKK1

dydt(26,1) = RF32 - RF33 - RF84;%cMEKK1_p38cyt
dydt(27,1) = -RF7 - RF8 - RF11 - RF14 + RF64 - RF127;%FL
dydt(28,1) = RF1 - RF2 - RF4 - RF7 - RF89-RF114;%DISC
dydt(29,1) = -RF1 + RF44;%FasL
dydt(30,1) = RF2 - RF3 - RF8 - RF9 - RF92-RF117;%DISC_pro8

dydt(31,1) = RF7 - RF10 - RF11 - RF12 - RF91-RF115;%DISC_FL
dydt(32,1) = -RF4 - RF9 - RF12 - RF15 + RF65 - RF128;%FS
dydt(33,1) = RF4 - RF13 - RF14 - RF15 - RF90-RF116;%DISC_FS
dydt(34,1) = 2*RF3 - 2*RF16 + RF18 - 2*RF28 - RF76;%p43p41
dydt(35,1) = RF8 + RF10 - RF19 - RF93;%p43_FLIP

dydt(36,1) = RF9 + RF13 - RF107-RF121;%DISC_pro8_FS
dydt(37,1) = RF11 - RF94-RF118;%DISC_FL_FL
dydt(38,1) = RF12 + RF14 - RF95-RF119;%DISC_FL_FS
dydt(39,1) = RF15 - RF96-RF120;%DISC_FS_FS
dydt(40,1) = -RF19 + RF66 - RF97;%IKK

dydt(41,1) = RF19 - RF22;%p43_FLIP_IKK_a
dydt(42,1) = -RF20 + RF67 - RF98;%NFkB_IkB
dydt(43,1) = RF20 - RF21 - RF99;%NFkB_IkB_p
dydt(44,1) = RF21 - RF23 - RF53;%NFkB_cyt
dydt(45,1) = RF26 - RF48 - RF75 -RF111;%TSP1_CD36_pp59

dydt(46,1) = RF36 - RF38 + RF39 - RF50 - RF86;%pp38nuc
dydt(47,1) = RF37 + RF56 - RF87;%p38nuc
dydt(48,1) = -RF50 + RF56 + RF69 - RF70;%Ptase_nuc
dydt(49,1) = RF50 - RF56 - RF102;%pp38nuc_Ptase
dydt(50,1) = RF40 - RF41;%FasLm_nuc

dydt(51,1) = -RF38 - RF103 + RF106;%NFkB_nuc
dydt(52,1) = RF38 - RF39 - RF104;%pp38_NFkB
dydt(53,1) = RF39 - RF105;%NFkB_p
```

```
%% parameter values
type = 'weighted';
params = zeros(54,1);
params(1,1) = 30;%Kon_dephos
params(2,1) = 1;%Kdephos
params(3,1) = 60;%Ki_casp3_XIAP
params(4,1) = 0.1;%Ktrsp_cyt_nuc
params(5,1) = 0.003;%Kinter

params(6,1) = 5;%Vmax_FasLm
params(7,1) = 0.5;%Km_FasLm
params(8,1) = 0.6;%Kexp_FasLm
params(9,1) = 0.5;%Ksyn_FasL
params(10,1) = 0.6;%Kexp_FasL

params(11,1) = 30;%Kon_Fas_FasL
params(12,1) = 30;%Kon_DISC_pro8
params(13,1) = 0.0001; %Ksyn_all
params(14,1) = 0.001;%Kdeg
params(15,1) = 30;%Kon_casp3_MEKK1

params(16,1) = 60;%Kp_p38
params(17,1) = 60;%Kc_MEKK1
params(18,1) = 30; % Kon_cMEKK1_p38
params(19,1) = 20;%FasLsyn_factor
params(20,1) = 669.33; %Kon_DISC_FL

params(21,1) = 0.01; %Kon_DISC_FS
params(22,1) = 0.59466; %Kp43p41
params(23,1) = 1000;%Kp43FLIP
params(24,1) = 887.51;%Kon_DISC2_FS
params(25,1) = 0.80444;%K_casp8

params(26,1) = 2.2498;%Kc_casp3
params(27,1) = 120.53;%Kr_casp8
params(28,1) = 0.72043;%Ka_IKK
params(29,1) = 358.82;%Kp_NFkBIkB
params(30,1) = 3.6842;%Ka_NFkB

params(31,1) = 0.022299;%Kp_p38
params(32,1) = 0.006418;%Kdeg_NFkBcyt
params(33,1) =0.0289;%Kdeg_casp8
params(34,1) =0.15029;%Kdeg_casp3
params(35,1) =30;%Kon_pp38_NFkB v4

params(36,1) = 0.001; %Kdeg_pp38cyt
params(37,1) =30;%Kon_TSP1_CD36
params(38,1) = 30;%Kon_TSP1_CD36_p59
params(39,1) = 60;%Kp_p59
params(40,1) = 60;%Kp_NFkB

params(41,1) = 30;%Kon_casp3_PARP
params(42,1) = 60; %Kc_PARP
params(43,1) = 0.0001;%Ksyn_Ptase
params(44,1) = 0.001;%Kdeg_Ptase
params(45,1) = 60;%Kc_casp8

params(46,1) = 0.03;%Kinser
params(47,1) = 1.2e-2;%koff
params(48,1) = 1.2e-2;%Koff_dephos
params(49,1) = 1.2e-2;%Koff_casp3_MEKK1
```

```
params(50,1)=1.2e-2 ;%Koff_cMEKK1_p38

params(51,1)= 1.2e-2;%Koff_pp38_NFkB
params(52,1)=1.2e-2 ;%Koff_TSP1_CD36
params(53,1)= 1.2e-2;%Koff_TSP1_CD36_p59
params(54,1)= 1.2e-2;%Koff_casp3_PARP

cytosol = 0.824;
nucleus = 0.118;
mc = 0.058;

%% Initial Concentrations
y(1)= 0.000; %TSP1
y(2) = 0.0332; %CD36
y(3) = 0;%TSP1_CD36
y(4) = 0.34;%p59
y(5) = 0;%TSP1_CD36_p59

y(6)= 0.0647;%pro8
y(7)= 0;%casp8
y(8) = 0.00144;%pro3
y(9) = 0;%casp3
y(10) = 0.15;%p38cyt

y(11) = 0;%pp38cyt
y(12) = 0.017;%Ptase_cyt
y(13) = 0; %pp38cyt_Ptase
y(14) = 0;%pp59_Ptase
y(15) = 0.17;%XIAP

y(16) = 1.7;%PARP
y(17) = 0; %casp3_PARP
y(18) = 0;%cPARP
y(19) = 0;%casp3_u
y(20) = 0;%FasL_cyt

y(21) = 0; %FasLm_cyt
y(22) = 0.0149; %Fas
y(23) = 0; %casp3_MEKK1
y(24) = 0; % cMEKK1
y(25) = 0.025; %MEKK1

y(26) = 0; %cMEKK1_p38cyt
y(27) = 0.0073986; % FL
y(28) = 0; %DISC
y(29) = 0;%FasL
y(30) = 0;%DISC_pro8

y(31) = 0;%DISC_FL
y(32) = 0.0050839;%FS
y(33) = 0;%DISC_FS
y(34) = 0;%p43p41
y(35) = 0;%p43_FLIP v4

y(36) = 0;%DISC_pro8_FS
y(37) = 0;% DISC_FL_FL
y(38) = 0; %DISC_FL_FS
y(39) = 0;%DISC_FS_FS
y(40) = 0.0057728;%IKK

y(41) = 0;%p43_FLIP_IKK_a
y(42) = 0.0047395;%NFkB_IkB
y(43) = 0; %NFkB_IkB_p
```

```
y(44) = 0.0008; %NFkB_cyt
y(45) = 0; %TSP1_CD36_pp59

y(46) = 0; %pp38nuc
y(47) = 0; %p38nuc
y(48) = 0.017; %Ptase_nuc
y(49) = 0; %pp38nuc_Ptase
y(50) = 0; %FasLm_nuc

y(51) = 0;%NFkB_nuc
y(52) = 0;%pp38_NFkB
y(53) = 0; %NFkB_p
```
