# Supplementary material for: Predictive model identifies strategies to enhance TSP1-mediated apoptosis signaling
Source: Cell Commun Signal. 2017 Dec 19;15:53. doi: 10.1186/s12964-017-0207-9 (PMC5735807; doi:10.1186/s12964-017-0207-9)
Supplement: Supplementary file 3 — Comparison of minimal model of FasL signaling to experimental data. Figure S2. Global sensitivity analysis of parameters. Figure S3. Global sensitivity analysis of non-zero initial concentrations. Figure S4. Receptor number distributions of CD36 and Fas. Figure S5. Distribution of estimated parameter values. Figure S6. Dynamics of all species in baseline model. Figure S7. Population-level response to 10 nM TSP1 stimulation. Figure S8. Population-level response to 0.1 nM TSP1 stimulation. (PDF 13089 kb) [file 12964_2017_207_MOESM3_ESM.pdf]

Supplemental Figures:

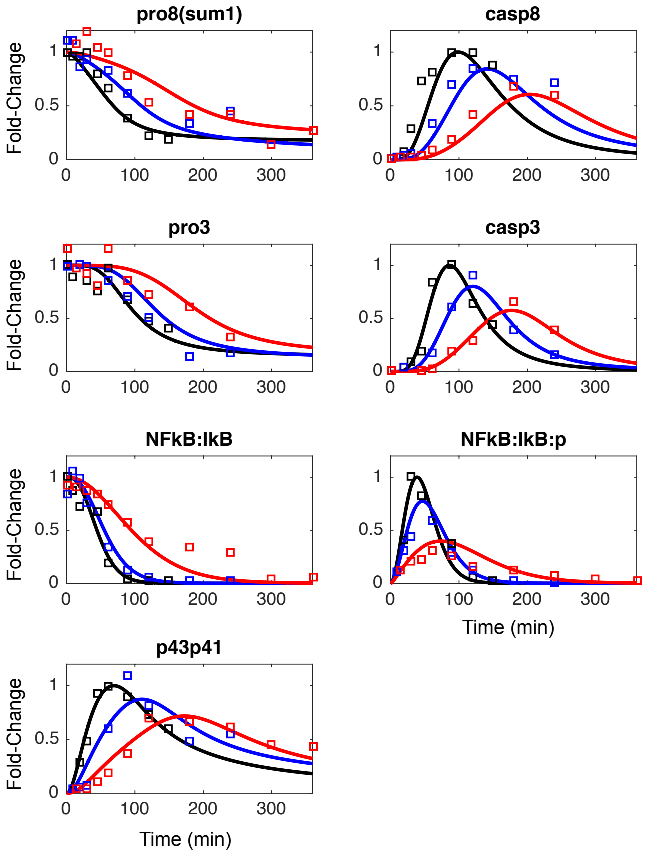

Figure S1. Comparison of minimal model of FasL signaling to experimental data. We implemented a minimal based on the work of Neumann *et al.* We included reversible reactions to mirror how other binding interactions are implemented in our full model. Predictions from the minimal model (lines) matches the original data (squares). FasL concentration used in model simulations: 1500ng/ml (black), 500ng/ml (blue), and 250ng/ml (red).

Figure S2

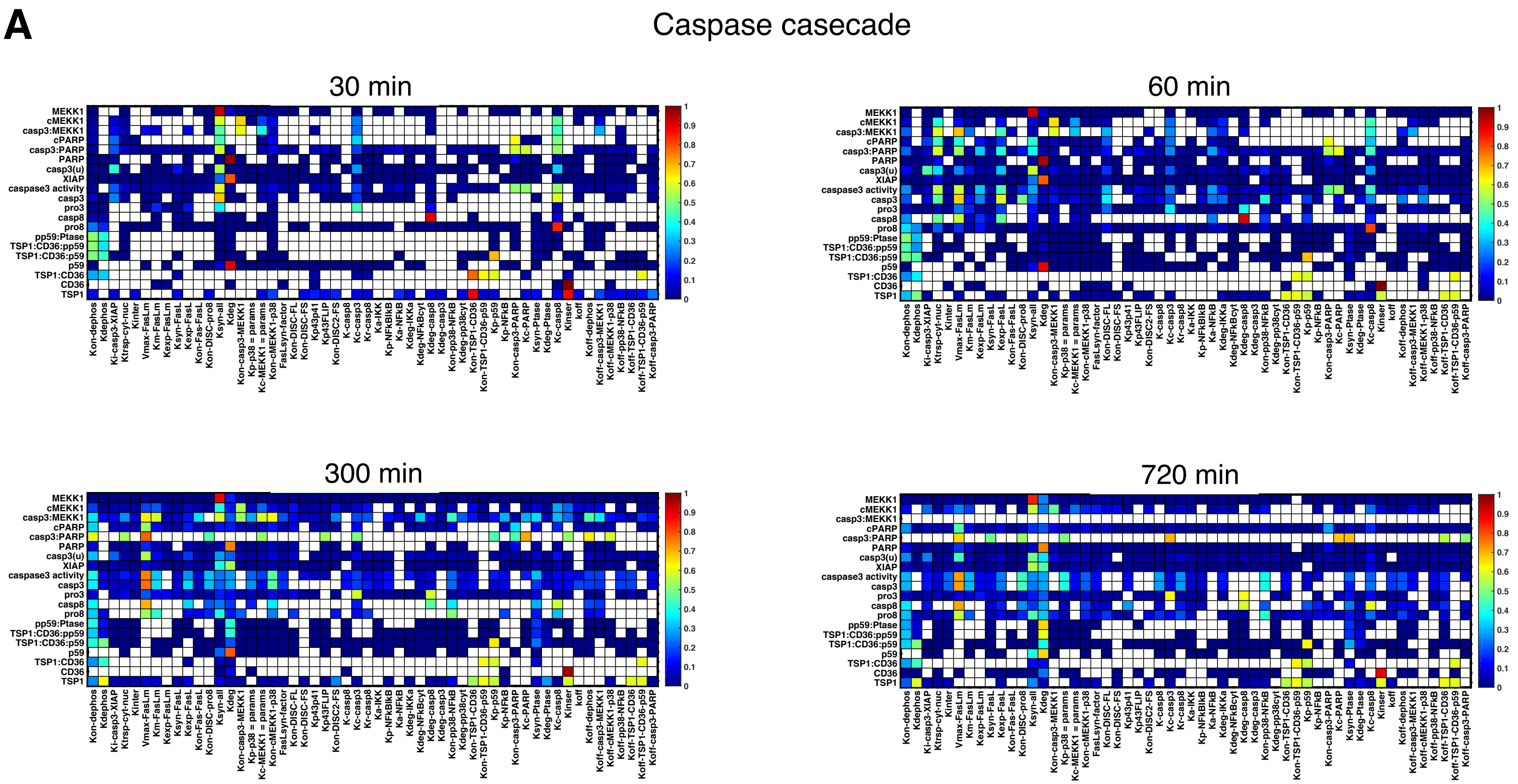
A
Caspase casecade
30 min
60 min
300 min
720 min
MEKK1
cMEKK1
casp3:MEKK1
cPARP
casp3:PARP
PARP
casp3(u)
XIAP
caspase3 activity
casp3
pro3
casp8
pro8
pp59:Ptase
TSP1:CD36:pp59
TSP1:CD36:p59
p59
TSP1:CD36
CD36
TSP1
Kon-dephos
Kdephos
Ki-casp3-XIAP
Ktrsp-cyt-nuc
Kinter
Vmax-FasLm
Km-FasLm
Kexp-FasLm
Ksyn-FasL
Kexp-FasL
Kon-Fas-FasL
Kon-DISC-pro8
Ksyn-all
Kdeg
Kon-casp3-MEKK1
Kp-p38 = params
Kc-MEKK1 = params
Kon-cMEKK1-p38
FasLsyn-factor
Kon-DISC-FL
Kon-DISC-FS
Kp43p41
Kp43FLIP
Kon-DISC2-FS
K-casp8
Kc-casp3
Kr-casp8
Ka-IKK
Kp-NFkBIkB
Ka-NFkB
Kdeg-IKKa
Kdeg-NFkBcyt
Kdeg-casp8
Kdeg-casp3
Kon-pp38-NFkB
Kdeg-pp38cyt
Kon-TSP1-CD36
Kon-TSP1-CD36-p59
Kp-p59
Kp-NFkB
Kon-casp3-PARP
Kc-PARP
Ksyn-Ptase
Kdeg-Ptase
Kc-casp8
Kinser
koff
Koff-dephos
Koff-casp3-MEKK1
Koff-cMEKK1-p38
Koff-pp38-NFkB
Koff-TSP1-CD36
Koff-TSP1-CD36-p59
Koff-casp3-PARP
0
0.1
0.2
0.3
0.4
0.5
0.6
0.7
0.8
0.9
1

Figure S2

B p38 signaling

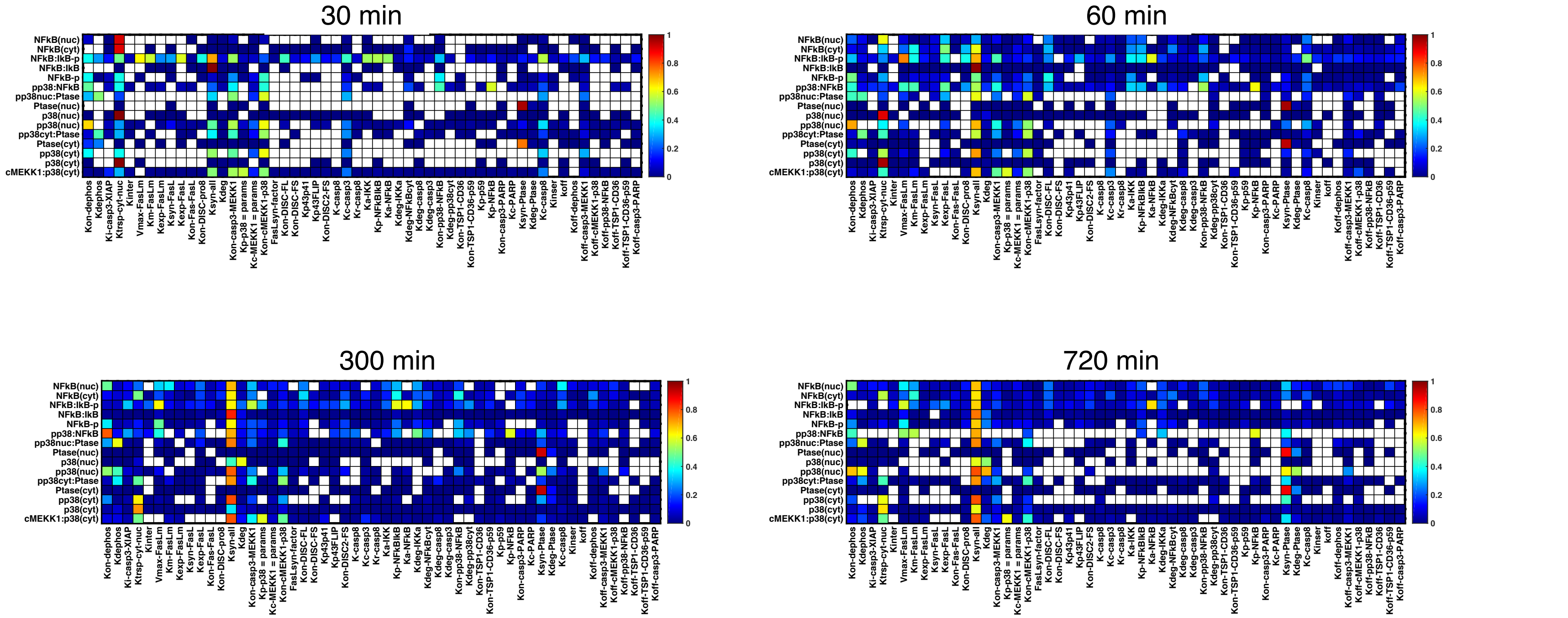

Figure S2

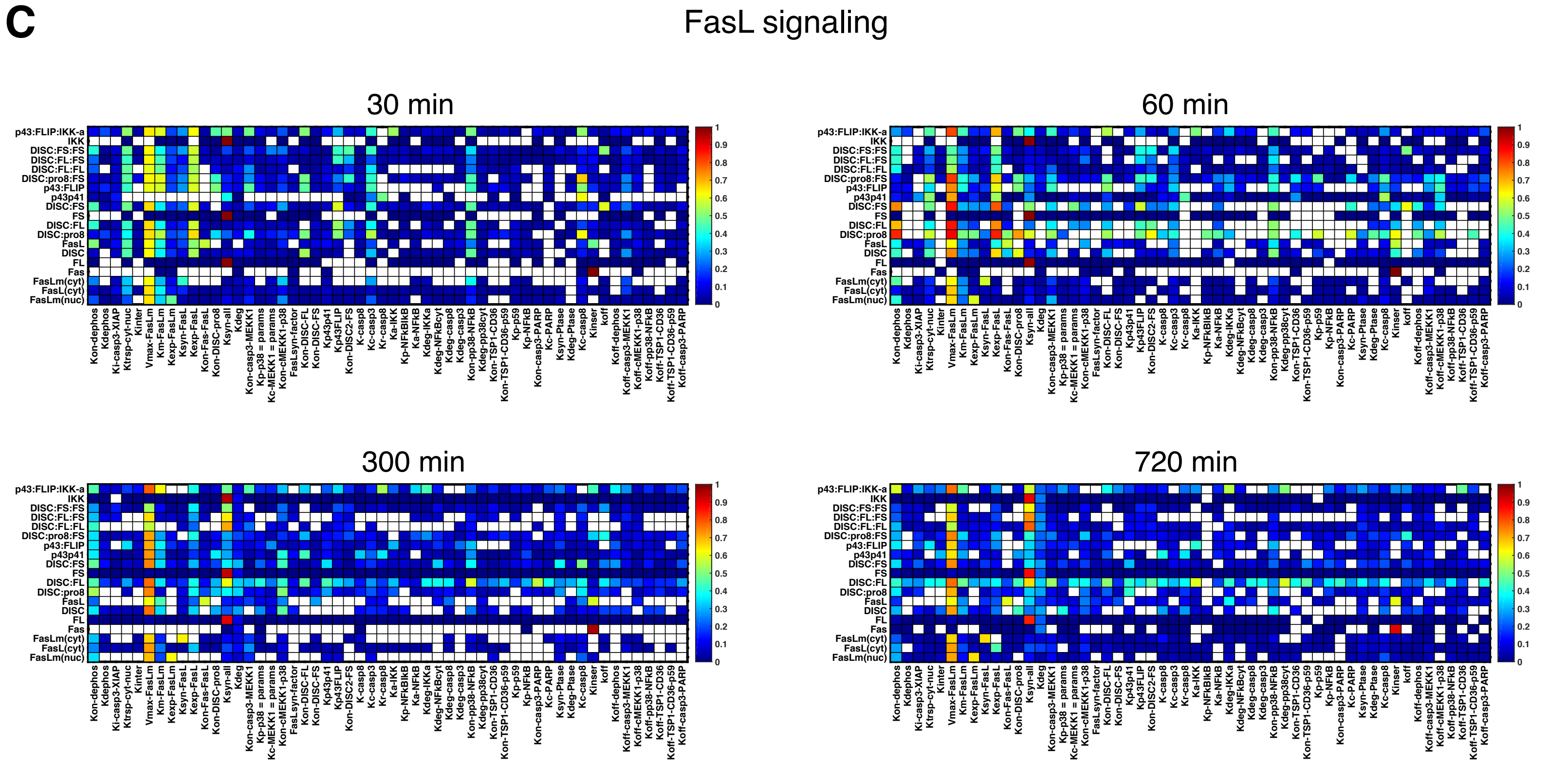

Figure S2. Global sensitivity analysis of parameters. An eFAST sensitivity analysis was performed to identify which parameter most significantly influence the cPARP concentration predicted by the model for different simulated time points. The total sensitivity index is shown for each parameter. *x*-axis: parameters (inputs); *y*-axis: model species (outputs). A) Parameters involved in upstream signaling (“Caspase cascade”); B) Parameters involved in intermediate signaling (“p38 signaling”); and C) Parameters involved in downstream signaling (“FasL signaling”).

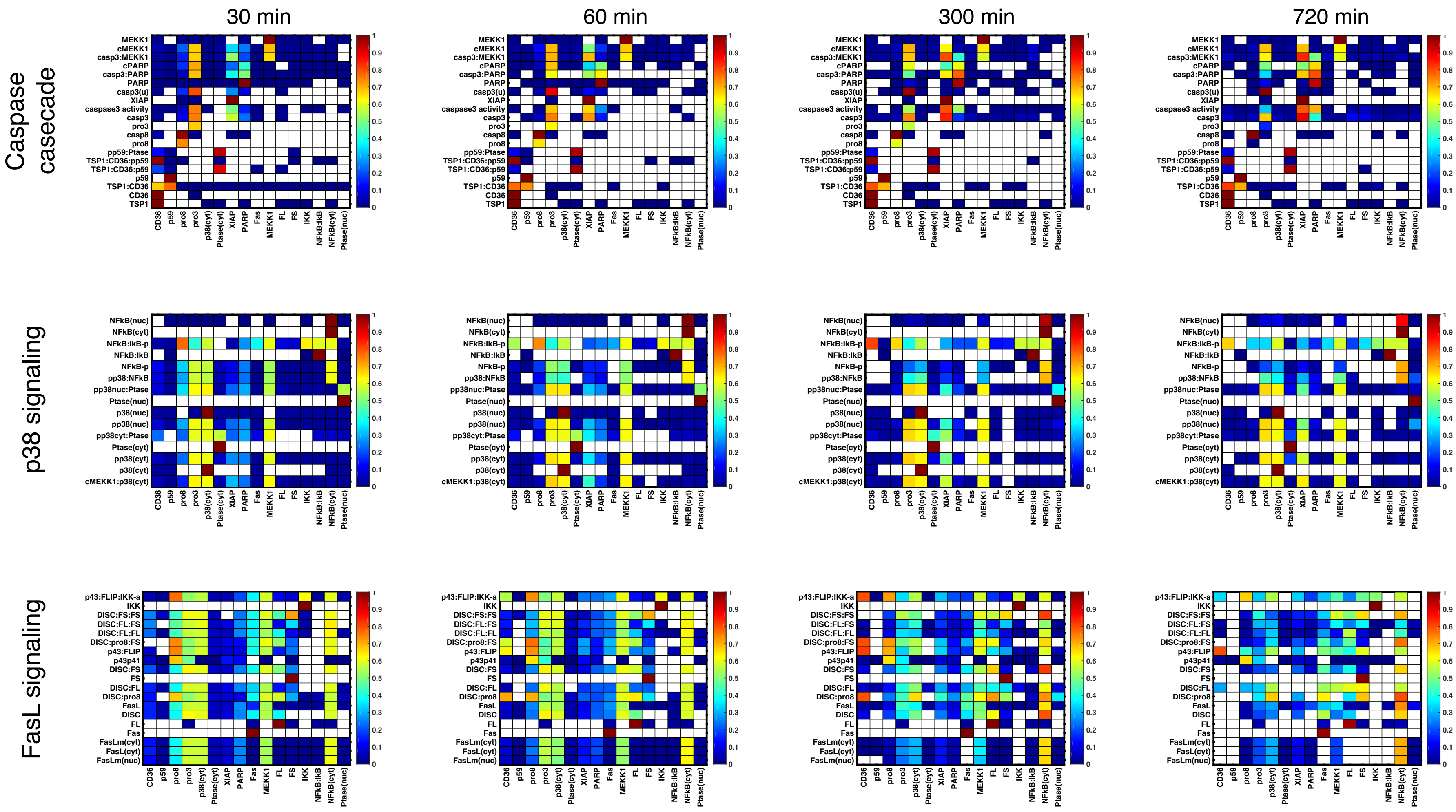

Figure S3. Global sensitivity analysis of non-zero initial concentrations. We performed eFAST sensitivity analysis to identify which initial concentrations most significantly influence the cPARP concentration predicted by the model for different simulated time points. The total sensitivity index is shown for each species with a non-zero initial condition. *x*-axis: non-zero initial concentrations (inputs); *y*-axis: model species (outputs). Plots are arranged by upstream ("Caspase cascade"), intermediate ("p38 signaling"), and downstream signaling ("FasL signaling").

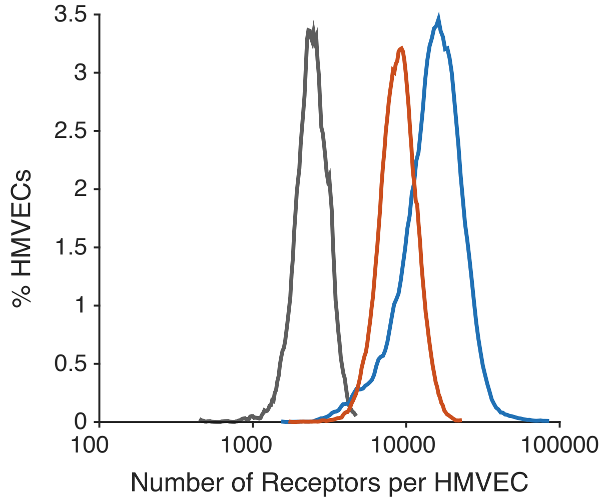

Figure S4. Receptor number distributions of CD36 and Fas. Histogram showing distributions of CD36 receptor (blue) and Fas receptor (red) on HMVECs. Two representative samples of each receptor quantification measurement are shown (a total of 12,286 cells for CD36 and 11,013 cells for Fas). Grey: unstained control (3,840 cells).

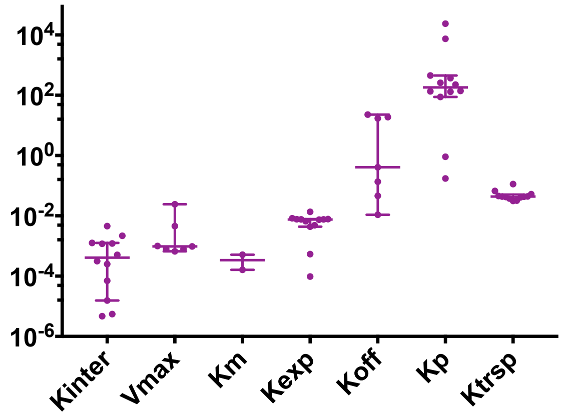

Figure S5. Distribution of estimated parameter values.

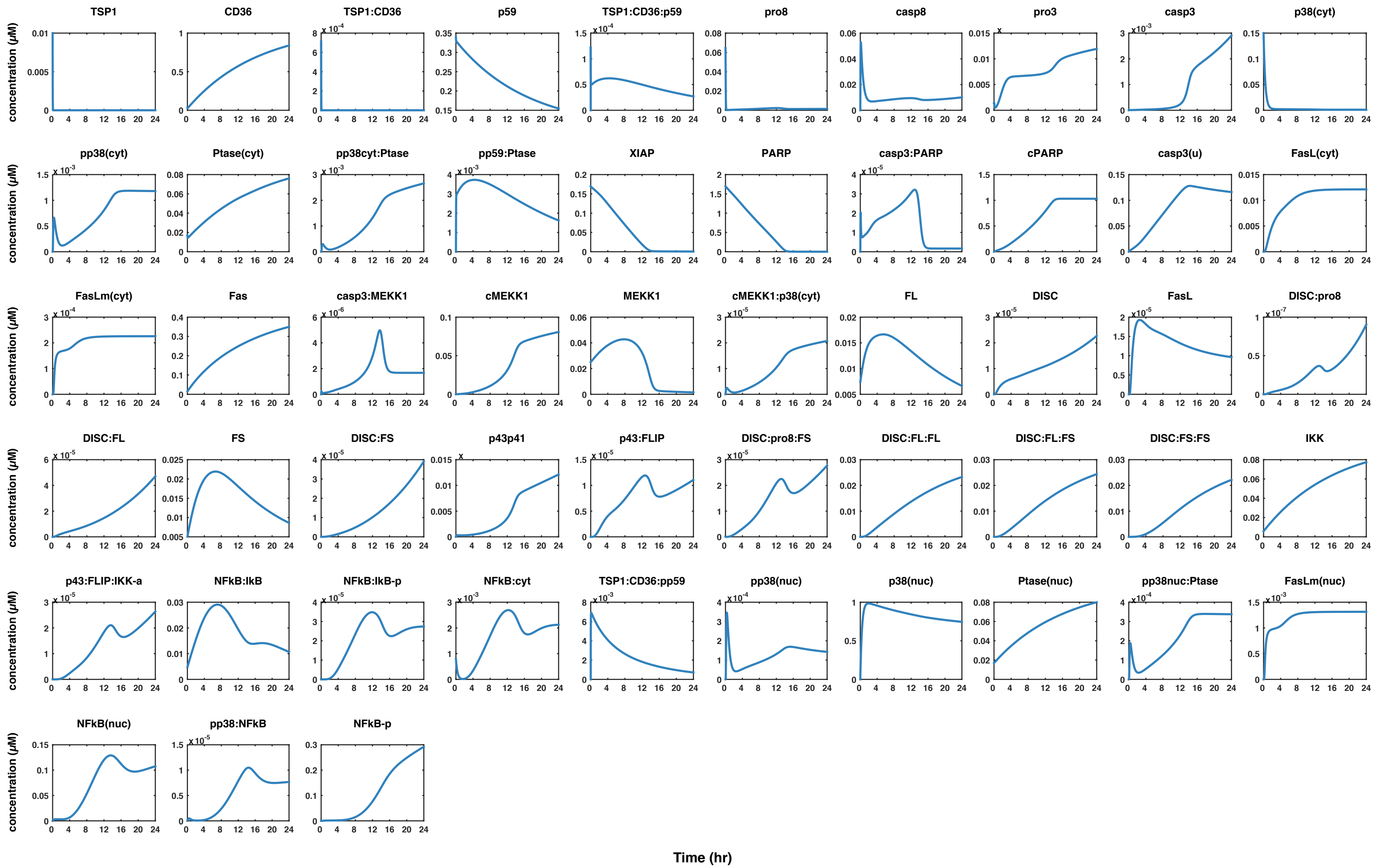

Figure S6. Baseline model simulated dynamics of all species with TSP1 stimulation. The concentrations of all 53 species in the modeled network with 10 nM TSP1 stimulation were simulated with the best set of parameter values.

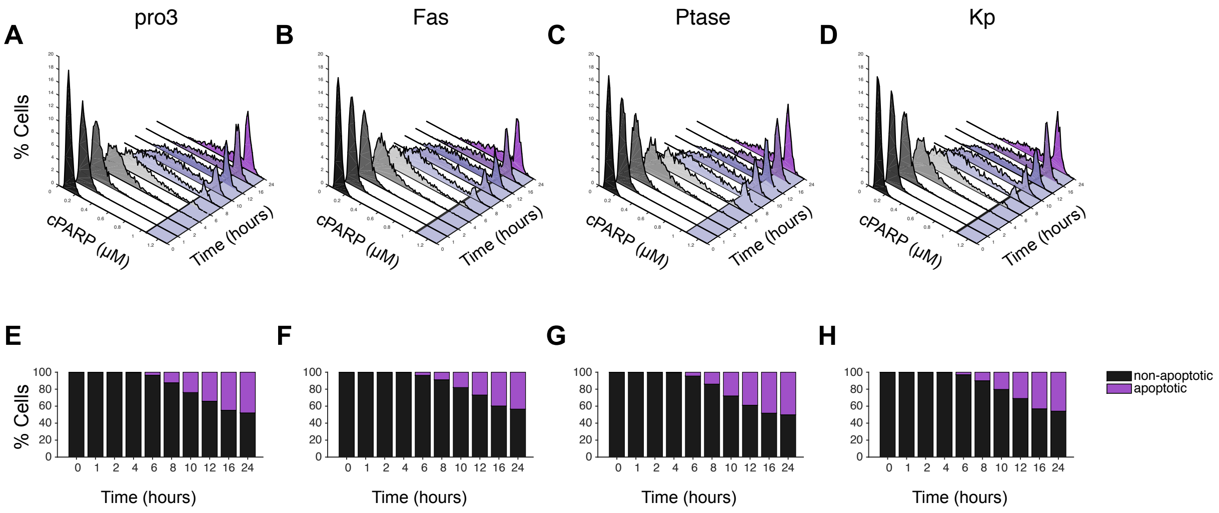

Figure S7. Population-level response to TSP1 stimulation. (A)-(D): Histogram showing the percentage of the 2,000 cells with a given cPARP concentration, in response to 10 nM TSP1 stimulation with A) procaspase-3 overexpression; B) Fas overexpression; C) phosphatase inhibition; and D) increasing kinase activity. A different color is assigned to each time point, and shading on the *x*-*y* plane indicates the threshold cPARP concentration for classifying cells as apoptotic (1.05 nM). (E)-(H): The predicted percentage of non-apoptotic (black) and apoptotic (purple) cells in response to 10 nM TSP1 stimulation with E) procaspase-3 overexpression; F) Fas overexpression; G) phosphatase inhibition; and H) increasing kinase activity.

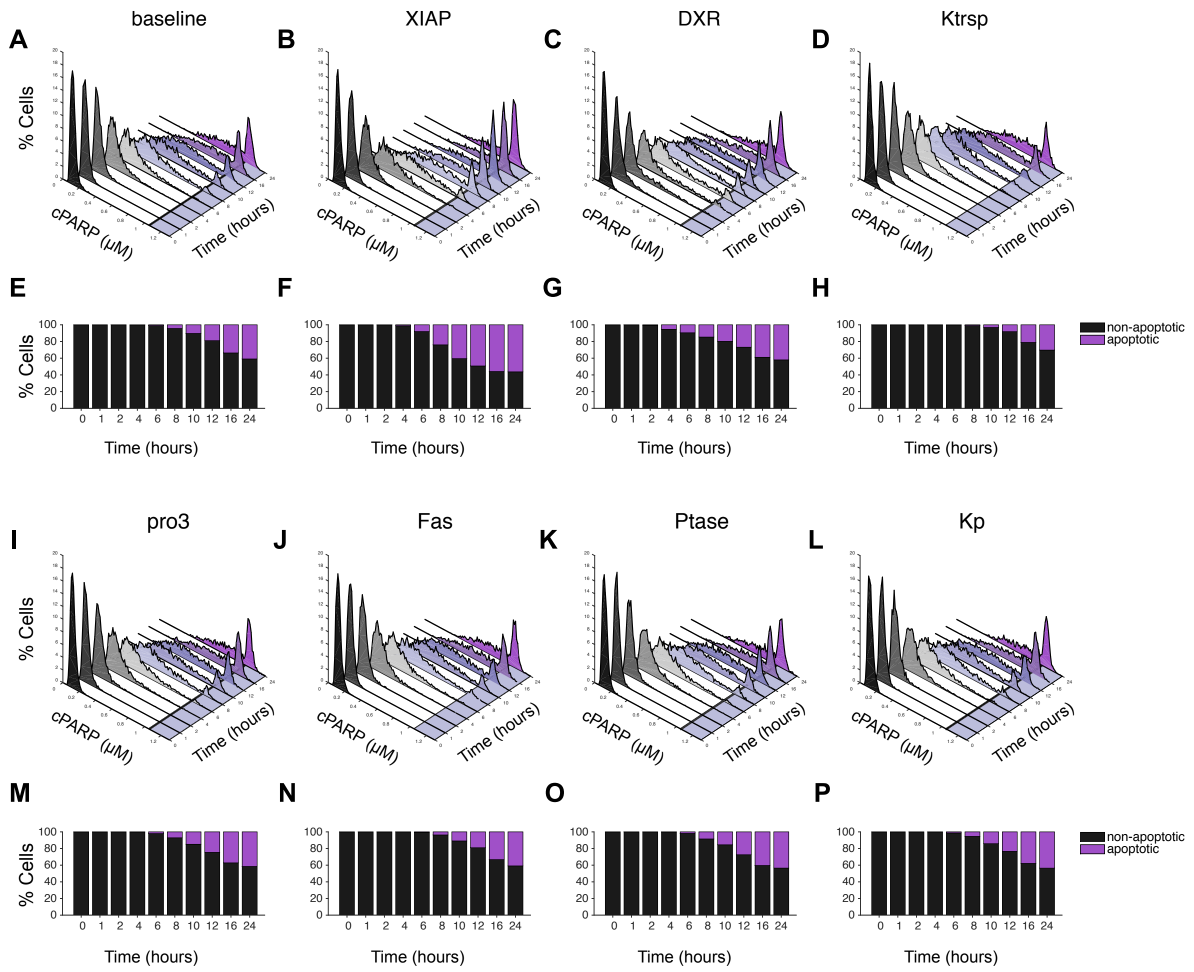

Figure S8. Population-level response to 0.1 nM TSP1 stimulation. (A)-(D): Histogram showing the percentage of the 2,000 cells in response to 0.1 nM TSP1 stimulation. A) Baseline model; B) XIAP downregulation; C) DXR treatment; and D) Increased nuclear translocation rate. A different color is assigned to each time point. (E)-(H): The predicted percentage of non-apoptotic (black) and apoptotic (orange) cells in response to 0.1 nM TSP1 stimulation. E) Baseline model; F) XIAP downregulation; G) DXR treatment; and H) Increased nuclear translocation rate. (I)-(L): Histogram showing the percentage of the 2,000 cells in response to 0.1 nM TSP1 stimulation with I) procaspase-3 overexpression; J) Fas overexpression; K) phosphatase inhibition; and L) increasing kinase activity. (M)-(P): The predicted percentage of non-apoptotic (black) and apoptotic (orange) cells in response to 0.1 nM TSP1 stimulation with M) procaspase-3 overexpression; N) Fas overexpression; O) phosphatase inhibition; and P) increasing kinase activity. In (A)-(D) and (I)-(L), shading on the *x*-*y* plane indicates the threshold cPARP concentration for classifying cells as apoptotic (1.05 nM).
